# Stem Cell-Derived Organoids for Cancer Therapy: Precision Medicine and Drug Selection

**DOI:** 10.3390/ijms27072954

**Published:** 2026-03-24

**Authors:** Md. M. N. Azim, Sujay Kumar Bhajan, Jun Hong Park, Kasim Sakran Abass, Atikur Rahman, Min Choi, Jinwon Choi, Sohyun Park, Hyo Jeong Kim, Salima Akter, Amama Rani, Bonglee Kim

**Affiliations:** 1 Department of Physiology, College of Veterinary Medicine, Jeonbuk National University, Iksan 54596, Republic of Korea; azimmdmn@jbnu.ac.kr (M.M.N.A.); jhpark77@jbnu.ac.kr (J.H.P.); 2 Department of Physiology, Biochemistry, and Pharmacology, College of Veterinary Medicine, University of Kirkuk, Kirkuk 36001, Iraq; kasim_abass@uokirkuk.edu.iq; 3 Department of Biotechnology & Genetic Engineering, Gopalganj Science & Technology University, Gopalganj 8100, Bangladesh; sujaybge@gstu.edu.bd; 4 Department of Animal Nutrition, Genetics and Breeding, Faculty of Animal Science and Veterinary Medicine, Sher-e-Bangla Agricultural University, Dhaka 1207, Bangladesh; atikurrahman.sau@gmail.com; 5 Department of Pathology, College of Korean Medicine, Kyung Hee University, Seoul 02447, Republic of Korea; chlals2078@khu.ac.kr (M.C.); 2022310848@khu.ac.kr (J.C.); shpark0912@khu.ac.kr (S.P.); hyojeong25@khu.ac.kr (H.J.K.); aktersalima@khu.ac.kr (S.A.); amama.rani@khu.ac.kr (A.R.); 6 Korean Medicine-Based Drug Repositioning Cancer Research Center, College of Korean Medicine, Kyung Hee University, Seoul 02447, Republic of Korea

**Keywords:** patient-derived organoids, cancer stem cells, precision oncology, drug screening, regenerative medicine, translational models

## Abstract

Millions of new cancer cases and deaths worldwide annually demonstrate the pressing need for predictive preclinical models that go beyond standard two-dimensional (2D) cultures and animal systems. Recent developments in three-dimensional (3D) organoid technology have yielded a powerful platform for generating patient-specific mini-organ models that faithfully recapitulate primary tumors at the genetic, phenotypic, and architectural levels. Organoids retain functional fidelity by preserving key stem cell signaling pathways, including Wnt, Notch, and Hippo, making them robust platforms for disease modeling and high-throughput drug screening. This review describes representative organoid systems, ranging from patient-derived organoids (PDOs) to induced pluripotent stem cell (iPSC)-derived organoids, that serve as disease-specific “avatars” for personalized therapeutics. Predictive accuracy rates greater than 90% have been shown in clinical studies, providing evidence for the relevance of organoids in functional precision medicine. In addition to drug discovery, the extended use of organoids in regenerative oncology can provide a unique regulatory mechanism by selectively targeting CSCs and enhancing tissue repair after cytotoxic treatments. Recent advances in organoid-on-a-chip platforms, 3D bioprinting, and artificial intelligence (AI) address critical challenges involving vascularization, immune system integration, and scalability. With the advent of standardized, GMP-compliant platforms and recent regulatory initiatives, such as the FDA Modernization Act 2.0, organoids are well-positioned to support next-generation cancer research and therapy. This review aims to bridge the gap between stem cell-derived organoids (SCDOs), providing a fully humanized platform for preclinical cancer modeling and their clinical application, and to discuss their potential to advance ethically guided, personalized cancer therapeutics with improved predictive and translational power.

## 1. Introduction

**Figure 1:**
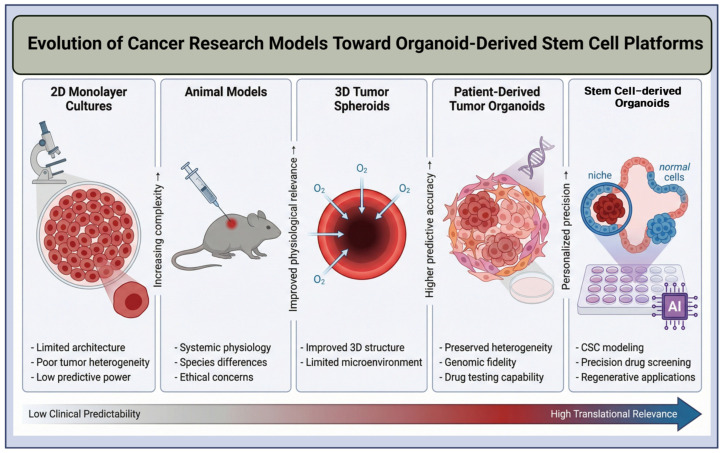
Evolution of cancer research models toward stem cell-derived organoid (SCOD) platforms. Schematic overview of the progression from 2D monolayer cultures and animal models to 3D tumor spheroids, patient-derived tumor organoids, and advanced organoid-derived stem cell platforms. The figure highlights increasing biological complexity, physiological relevance, predictive accuracy, and translational potential, culminating in precision drug screening, cancer stem cell modeling, and regenerative applications. The gradient bar indicates the transition from low clinical predictability to high translational relevance. Created with BioRender.com (version 2026); Jang, H. (2026) https://BioRender.com/o13vyea (accessed on 1 March 2026).

Cancer is one of the most pressing global health issues, with nearly 20 million new cases and 9.7 million deaths worldwide in 2022 [1]. Statistical analysis predicts that annual cancer deaths will rise to 13.2 million by 2030 [2]. Although cancer treatment has advanced significantly in recent years, cancer management remains challenging because of systemic toxicity and lack of selective therapy, which can lead to patient death during chemotherapy and radiotherapy [3]. Additionally, drug resistance and intratumor heterogeneity make it difficult to use the same therapies for all patients, as cancer cells vary in their sensitivity to medications by cell type [4]. One of the major constraints in developing effective cancer treatments is the need for predictive clinical models that can accurately mimic a patient’s condition [5]. The traditional 2D cell culture model fails to reproduce the complex microenvironment and organ-like 3D organization [6]. As a result, drugs that show promising results in 2D culture become ineffective or toxic when used in clinical trials. Xenografts in animal models are effective and widely used, but they are expensive, hard to maintain, and raise ethical concerns [7]. However, due to species differences, xenograft models also fail to mimic human physiology and the immune system, which significantly hamper oncology drug trials [8]. Therefore, a critical challenge remains to develop human-mimicking, physiologically relevant models for cancer drug trials & biomarker discovery (Figure 1). In that case, 3D organoid culture technology may offer a promising solution to overcome the limitations of 2D cultures and animal models [9,10]. Organoids are small, self-organizing 3D structures derived from adult stem cells (ADSCs), Embryonic Stem Cells (ESCs), or iPSCs [11]. Unlike 2D cell culture, they don’t form a monolayer. Instead, they form a multilayer structure that captures the structural features and genetic and phenotypic properties of the tissue from which they were derived, including histological structure and the mutational profile of primary tumors. Thus, they produce physiologically relevant ex vivo models for studying human development and disease [12]. Organoids can be produced from both normal stem cells and cancer stem cells. Normal stem cells produce a 3D organ-like structure, and CSCs produce tumors similar to the cancer origin. Organoid technology enables CSCs’ self-renewal and differentiation while recapitulating the ordered organization of the primary tumor. As CSCs drive tumor growth, metastasis, and therapeutic resistance, organoid models derived from these cells provide a valuable platform for evaluating anticancer therapeutics, studying tumor biology and drug resistance, and detecting predictive biomarkers in cancer [13,14].

Patient-derived organoids (PDOs) derive from numerous cancer types, such as colorectal, breast, gastric, and lung cancer. They can contain patients’ genomic profiles and treatment responses, serve as “living biobanks”, which may bring revolutionary change in oncology [15]. Moreover, genomic profiling often fails to reliably predict clinical drug responses, whereas organoid platforms enable large-scale drug screening and sensitivity testing to facilitate functional precision medicine [16]. This strategy can also predict response to chemotherapy and determine response-adapted cancer therapeutics with minimal toxicity and maximum efficacy. In addition to conventional chemotherapy and targeted therapies, emerging therapeutic strategies such as nanomedicine, photodynamic therapy, photothermal therapy, and nanoparticle-based drug delivery systems are increasingly being explored for precision cancer treatment. These technologies enable targeted delivery, controlled drug release, and selective tumor destruction while minimizing systemic toxicity. Advanced preclinical models, including organoid platforms, provide an important opportunity to evaluate the efficacy, safety, and tumor-specific responses of these novel therapeutic modalities in a physiologically relevant microenvironment.

This review highlights the paradigm shift in which SCDOs transform cancer drug discovery and personalized regenerative medicine, discussing the biological basis of diverse stem cell sources; emerging organoid-on-a-chip technologies; and the use of CRISPR gene editing and AI to improve model fidelity, reproducibility, and clinical translation. Collectively, the evidence delineates organoid technology as a transformative tool in the new era of predictive, personalized, and regenerative oncology, bridging experimental and patient-oriented research.

## 2. Biological Foundations of Stem Cell-Derived Organoids

All stem cells possess an innate developmental program that enables them to spontaneously organize into complex 3D structures that mimic the architecture and cellular composition of the tissues from which they are derived [17]. These programs are coordinated at the stem cell niche by tightly regulated, evolutionarily conserved signaling pathways that dictate cellular polarity, lineage specification, spatial organization, and tissue morphogenesis [18]. With the developmental plasticity of SCODs, it is possible to form organotypic models that recapitulate in vivo physiology and provide disease-appropriate platforms with tissue homeostasis for cancer research.

### 2.1. In Vitro Regeneration of the Stem Cell Niche

Successful in vitro reconstruction of the stem cell niche is a crucial step for organoid development, maintenance, and large-scale expansion [19]. The endogenous niche of a stem cell comprises soluble growth factors, membrane-tethered ligands, components of the extracellular matrix (ECM), and biomechanical signals, which together guide stem cell fate decisions [20]. These signals must be controlled to support proliferation, self-renewal, lineage commitment, and tissue architecture throughout an organoid program. The control of these signals has led to the development of sophisticated biomaterials that replicate the chemical and mechanical properties of native ECM, examples being tunable hydrogels. Beyond its structural scaffolding role, the ECM serves as the master regulator of cell adhesion, polarity, and intracellular signaling cascades [21]. Nevertheless, soluble growth factors and cytokines promote mitogenic and adhesive pathways, while the biomechanical properties of the adjacent matrix carefully adjust their effectiveness. Through mechanotransduction, stem cells perceive matrix-derived mechanical cues, such as stiffness, topography, and tensile forces, which are translated to biochemical signals that regulate transcriptional networks involved in the homeostasis of multipotency and differentiation [22]. Together, this coupling of biochemical signaling with anisotropic mechanical forces orchestrates cellular contraction, division, and spatial patterning to ensure organoid morphogenesis and structural integrity despite the disorder of 3D cultures [23].

### 2.2. Core Signaling Pathways of Organoid Patterning

**Figure 2:**
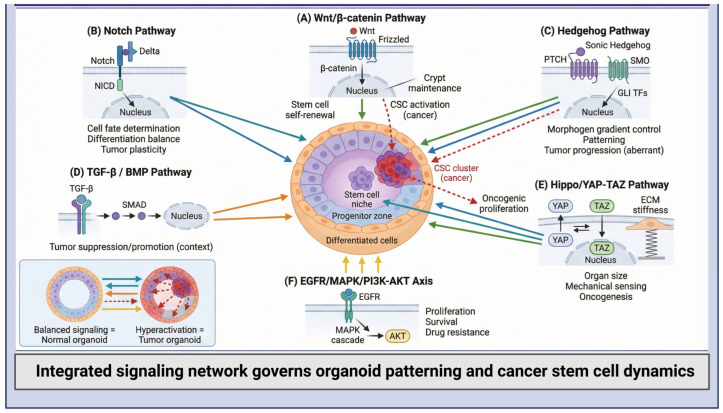
Key signaling pathways governing organoid development and cancer progression. Comprehensive schematic illustrating the principal signaling networks that regulate stem cell homeostasis, lineage specification, and tumorigenesis within organoid systems. (**A**) Wnt/β-catenin signaling maintains stem cell self-renewal and crypt architecture under physiological conditions, whereas constitutive activation drives CSC expansion and tumor initiation. (**B**) Notch signaling orchestrates cell-fate commitment, the differentiation equilibrium, and tumor cell plasticity. (**C**) Hh signaling regulates morphogen gradient formation and spatial tissue patterning, with aberrant activation contributing to malignant progression. (**D**) TGF-β/BMP signaling mediates context-dependent tumor-suppressive or tumor-promoting effects through SMAD-dependent transcriptional regulation. (**E**) Hippo/YAP–TAZ signaling integrates biomechanical cues, including ECM stiffness, to control organ size, proliferation, and oncogenic transformation. (**F**) The EGFR/MAPK/PI3K–AKT axis promotes cellular proliferation, survival, metabolic adaptation, and therapeutic resistance. Created with BioRender.com (version 2026); https://BioRender.com/4i2ea3r (accessed on 1 March 2026).

Complex networks of signaling pathway interactions operate in a context-dependent manner to direct the assembly, spatial organization, and differentiation of organoids [24]. They coordinate stem cell maintenance, fate decisions, and tissue architecture across multiple scales to influence organoid formation, structure, and function. The balance of pathway activation and inhibition, along with inter-pathway crosstalk, determines the fate of stem cell populations (Figure 2).

Wingless/Integrated (Wnt) signaling is a master regulator of stem cell proliferation and maintenance, maintaining this long-term self-renewing compartment, promoting constant expansion, and preventing premature differentiation [25]. Aberrant activation of Wnt signaling has been reported in several malignancies, particularly colorectal cancer, hepatocellular carcinoma, and breast cancer, where it contributes to uncontrolled proliferation, tumor initiation, and the maintenance of cancer stem cell populations [26]. Hence, specific modulation of Wnt gradients is important for maintaining organoid structural integrity and preserving tissue-specific stem cell hierarchies. Another signaling pathway mediating direct cell-to-cell communication is Notch, which regulates cell fate decisions by balancing stem cell maintenance and differentiation in response to multiple contextual signaling cues [27]. Dysregulation of the Notch pathway has been implicated in a variety of cancers, including T-cell acute lymphoblastic leukemia (T-ALL), breast cancer, pancreatic cancer, and colorectal cancer, where abnormal signaling can promote tumor progression, influence cancer stem cell maintenance, and contribute to therapy resistance [28,29]. Notch signaling maintains appropriate cellular composition and organizational hierarchy during organoid development. Through lateral inhibition and context-responsive activation, it generates spatially organized domains while regulating the balance between stem cell maintenance and differentiation. Another major pathway is the evolutionarily conserved Hedgehog (Hh) pathway, which regulates embryonic morphogenesis and adult tissue organization [30]. Activation of the Hh pathway in an uncontrolled manner drives oncogenesis, in part by maintaining cancer stem cells and tumor growth, indicating a potential therapeutic role for this pathway in neoplasia [31]. Additional pathways, such as the Transforming Growth Factor–beta (TGF-β)/Bone Morphogenetic Protein (BMP), serve as one of the key progenitor gatekeepers to control uncontrolled proliferation while initiating lineage specification [32]. During the early phase of tumorigenesis, TGF-β signaling acts as a regulator, preventing cell cycle progression and promoting tissue homeostasis. Conversely, at late-stage cancer, this pathway is hypothesized to play a pro-tumorigenic role by promoting invasion, metastasis, and immune evasion through complex crosstalk with additional oncogenic signaling networks [33]. Such functional duality underscores the complex, context-dependent regulatory mechanisms that control stem cell behavior in both normal and pathological conditions.

Another important organ size controller is the Hippo signaling pathway, named after the Drosophila Hippo gene [34]. Its downstream signaling effectors are Yes-Associated Protein (YAP) and Transcriptional co-activator with PDZ-binding motif (TAZ), mechanosensitive transcriptional coactivators able to sense variations in ECM, potentially integrating them in nuclear gene expression programs governing proliferation, apoptosis, and stemness [35]. Dysregulated Hippo/YAP signaling, through constitutive YAP/TAZ activation, is intimately associated with tumor initiation and progression of many solid tumors, underscoring the importance of its modeling in organoid systems [36]. These pathways create interrelated signaling networks that are context-dependent and regulate organoid self-assembly, differentiation pathways, and spatial organization through mutual interactions. The finesse of these pathways is critical for the development of reproducible, physiologically relevant organoid cultures [37]. Moreover, knowledge of their crosstalk is important for understanding cancer development and progression, as well as for predicting treatment response. Additional cancer-associated pathways, including phosphoinositide 3-kinase (PI3K)/AKT/mammalian target of rapamycin (mTOR)**,** Janus kinase/signal transducer and activator of transcription (JAK/STAT)**,** mitogen-activated protein kinase/extracellular signal-regulated kinase (MAPK/ERK)**,** and nuclear factor kappa B (NF-κB)**,** also govern organoid development [38]. Table 1 summarizes the major signaling pathways regulating organoid biology, their roles in cancer pathogenesis, and key in vitro niche modulators.

## 3. Organoid Platforms in Cancer Research

**Figure 3:**
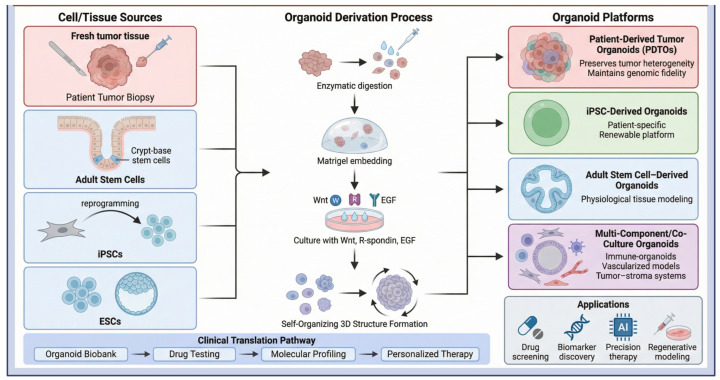
Cancer organoid platforms and derivation strategies. Schematic of organoid generation from patient tumor biopsies, adult stem cells, iPSCs, and ESCs. Following enzymatic digestion, embedding in extracellular matrix, and culture with niche factors (Wnt, R-spondin, EGF), cells self-organize into 3D organoids. Resulting platforms include patient-derived tumor organoids (PDTOs), iPSC-derived organoids, adult stem cell-derived organoids, and multi-component co-culture systems. The lower panel highlights translational applications, including biobanking, drug testing, molecular profiling, and personalized therapy. Created with BioRender.com (Version 2026); https://BioRender.com/dr5jqjw (accessed on 1 March 2026).

The development of various organoid platforms has enhanced the landscape of cancer research by enabling refined modeling of tumor biology, drug screening, and the implementation of precision medicine strategies. These models overcome the limitations of 2D cultures and animal models by recapitulating the complex in vivo tumor environment and genetic heterogeneity (Figure 3) (Table 2).

### 3.1. Patient-Derived Tumor Organoids

One notable achievement is the development of patient-derived tumor organoids (PDTOs) for personalized cancer therapy. These are 3D in vitro systems directly obtained from patient tumor samples, including primary surgical resections, metastatic sites, and small biopsy specimens [59]. So, organoids faithfully recapitulate intratumoral heterogeneity, preserving the diverse cellular subpopulations and molecular signatures characteristic of the patient’s primary tumor [60]. Notably, PDTOs uniquely combine genetic fidelity with preservation of tumor architecture and cellular diversity, making them particularly valuable for translational cancer research and drug response studies [61]. By preserving genomic and transcriptomic fidelity, PDTOs serve as patient-specific “avatars” for high-resolution profiling of drug sensitivity and resistance landscapes. Such precise modeling can predict clinical outcomes and help to pick the best treatment, thereby bridging experimental oncology with real-world care.

### 3.2. Induced Pluripotent Stem Cell (iPSC)-Derived Organoids

iPSC-derived organoids derive from somatic cells that can differentiate into nearly all somatic cell lineages, enabling indefinite in vitro expansion [19]. They have significant practical advantages, as they eliminate the need for repeated tissue biopsies and enable the scalable production of genetically matched, patient-specific material. So, they are used to model early tumorigenesis, hereditary cancer syndromes, and the functional consequences of specific genetic alterations in cancer development [62]. However, Researchers can introduce or correct specific mutations to model stepwise oncogenesis within a controlled genetic context. Moreover, iPSC-derived models are also helpful for modelling in sample-limited contexts, such as early developmental oncology or rare tumor types. Furthermore, iPSC-derived organoids also provide a robust platform for drug screening and biomarker discovery, offering a cost-effective, scalable, and reproducible system that recapitulates key aspects of organ-specific microenvironments in a species-independent manner [63].

### 3.3. Adult Stem Cell (ASC)-Derived Organoids

Organoids derived from ASCs originate from tissue-resident stem cell populations and retain the special functions of their original tissue [64]. Adult stem cells used for organoid generation can be isolated from multiple tissue sources, including intestinal crypts, liver, pancreas, bone marrow, and adipose tissue, as well as other epithelial tissues, reflecting the widespread distribution of tissue-resident regenerative stem cell niches in the human body [65,66,67]. So, they are indispensable tools for investigating normal tissue homeostasis, regeneration, and disease pathogenesis, as they retain tissue-specific functions. Importantly, ASCs can be genetically engineered to introduce cancer-driving mutations in a controlled manner, allowing reconstruction of physiologically relevant oncogenic progression in authentic 3D tissue. In addition, ASC-derived organoids are excellent preclinical models for drug screening assays. Moreover, ASC organoids can be proliferated, cryopreserved, and derived from patient samples, thereby empowering live biobanks that retain tissue-specific architecture and molecular profiles [68]. Such biobanks are important for translational research and personalized therapeutic approaches.

### 3.4. Co-Culture and Multi-Component Organoid Structures

Although monoculture organoids improved cancer modeling, the inherent biological complexity of tumors necessitates incorporating numerous tumor microenvironment (TME) components. To recap the multicellular and extracellular interactions that regulate tumor behavior, therapeutic responses, and disease progression, co-culture systems and multi-component organoid platforms have been established [69]. By integrating stromal, immune, and vascular components, these platforms enhance physiological relevance and boost the predictive accuracy of preclinical models (Table 3).

#### 3.4.1. Immune-Organoids

Immune organoids mix immunocompetent immune cells with tumor organoid cultures, an important consideration for anticancer immunotherapy research [73]. The TME includes various subtypes of immune populations, including macrophages, T lymphocytes, natural killer (NK) cells, and dendritic cells. Together, they are involved in tumor development, escape from host immune attack, and response to therapy. Tumor organoids can be co-cultured with autologous or allogeneic immune cells for immunotherapeutic targeting studies, including immune checkpoint blockade, chimeric antigen receptor (CAR) T cell-targeted cytotoxicity, and immune resistance mechanisms [74] (Table 3). These models help us examine the relationship between tumors and immune cells, thus enabling us to design next-generation immunotherapies most effectively.

#### 3.4.2. Vascularized Organoids

Conventional Organoids lack vascularization, which impedes oxygen and nutrient diffusion to the center of large organoids, hindering studies of metastasis, angiogenesis, and drug penetration [75]. A strategy to overcome this problem is the use of vascularized organoids, in which endothelial cells or mesodermal progenitors can spontaneously develop into capillary-like networks. More sophisticated strategies, such as 3D bioprinted vascular constructs and organ-on-a-chip platforms with perfusable microvasculature, can overcome these limitations [76]. These innovations enable modeling of systemic drug distribution, tumor angiogenesis, and tumor–circulatory system interactions, thereby substantially improving the translational utility of organoid-based drug screening platforms.

#### 3.4.3. Tumor–Stroma Models

The tumor stroma, comprising cancer-associated fibroblasts (CAFs), endothelial cells, and ECM components, plays a pivotal role in tumor growth, invasion, metastasis, and therapeutic resistance [77]. Tumor–stroma organoid models aim to replicate these interactions by co-culturing tumor organoids with defined stromal cell populations. For example, co-culture with fibroblasts can reveal how stromal cells establish tumor-permissive niches, often enriched in Wnt ligands, that promote cancer progression [78]. Such models provide valuable insight into the bidirectional communication between malignant cells and their microenvironment, uncover potential therapeutic targets within the non-malignant stroma, and offer strategies to disrupt pro-tumorigenic support systems.

## 4. Organoid-Based Models in Cancer Biology and Stem Cell Dynamics

The advent of SCDO platforms has revolutionized cancer research by moving from static tumor profiling to the dynamic modeling of tumor biology. This platform gives us an opportunity to learn how normal stem cells are transformed into cancerous states, as well as how cancer stem cells maintain tumor diversity and preserve architecture over time. These platforms facilitate high-throughput screening in tissue mimics and help identify what truly drives cancer growth.

### 4.1. Modeling Tumor Initiation and Progression

**Figure 4:**
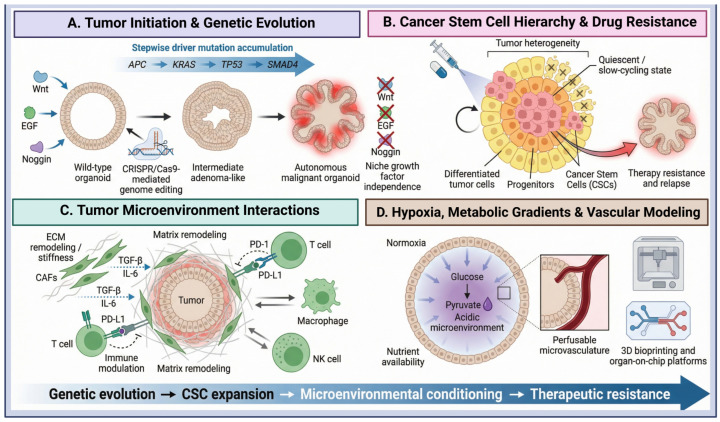
Organoid-derived stem cell platforms modeling tumor evolution and microenvironmental dynamics. Schematic diagram illustrating: (**A**) Sequential driver mutations (APC → KRAS → TP53 → SMAD4), introduced by CRISPR/Cas9, transform growth factor-dependent wild-type organoids into autonomous malignant organoids, recapitulating tumor initiation. (**B**) Tumor organoids exhibit a cancer stem cell (CSC) hierarchy, in which self-renewing CSCs generate progenitor and differentiated cells; persistence of CSCs under therapy drives relapse. (**C**) Co-culture with stromal and immune components models extracellular matrix (ECM) remodeling, cytokine signaling (TGF-β, IL-6), and PD-1/PD-L1/PD-L1-mediated immune modulation. (**D**) Oxygen and nutrient gradients establish hypoxic, metabolically reprogrammed niches, further enhanced by integration of perusable vasculature and organ-on-chip platforms. Created in BioRender.com (Version 2026); https://BioRender.com/2zpmx4f (accessed on 1 March 2026).

Non-cancerous tissue-derived organoids provide a flexible platform to recapitulate stepwise tumorigenic processes through accurate genetic manipulation. The adenoma-carcinoma sequence of Vogelstein’s colorectal cancer model is faithfully recapitulated by CRISPR/Cas9-mediated induction of mutations in Adenomatous Polyposis Coli (APC), Kirsten Rat Sarcoma Viral Oncogene Homolog (KRAS), Tumor Protein p53 (TP53), and SMAD Family Member 4 (SMAD4), also known as Deletion in Pancreatic Carcinoma 4 (DPC4) into organoids [79] (Figure 4A). These and other similar systems in which oncogenic mutations occur in a stepwise manner serve as models of escape from niche growth factor dependence, such as Wnt, Epidermal Growth Factor (EGF), and Noggin (a Bone Morphogenetic Protein antagonist), resulting in conversion of extrinsic stem cell regulation into a malignant state [80]. Non-tumorigenic tissue-derived organoids provide a flexible basal model to reconstitute stepwise tumorigenesis through targeted genetic manipulation. The adenoma-carcinoma sequence in the Vogelstein model for colorectal cancer is reproduced exactly when mutations are introduced by CRISPR/Cas9 into organoids, such as Adenomatous Polyposis Coli (APC), Kirsten Rat Sarcoma Viral Oncogene Homolog (KRAS), Tumor Protein p53 (TP53) and SMAD Family Member 4 (SMAD4) also known as Deletion in Pancreatic Carcinoma 4 (DPC4) [81]. As a result, Sequential oncogenic lesions in such models confer tumor resistance to niche growth-factor requirements for Wnt, Epidermal Growth Factor (EGF), and the antagonist of Bone Morphogenetic Protein, Noggin, thereby tuning extrinsic stem-cell regulation into a malignant property (Figure 4A) [82]. Organoid biobanking is not limited to colorectal cancer. It can also recapitulate early oncogenic features across various tissues. For example, normal human intestinal organoids have been genetically reprogrammed into invasive tumoroids mirroring advanced colorectal carcinogenesis, as well as cerebellar progenitor organoids harboring mutations in Patched 1 (PTCH1), which mimic the main molecular and histopathological profiles of medulloblastoma [83,84]. Interestingly, while driver-mutation-containing organoids do develop adenomas in vivo, full-fledged tumorigenesis also requires stromal and immune interactions that are absent in epithelial-only cultures, necessitating a co-culture approach for physiological relevance [85].

### 4.2. Cancer Stem Cells (CSCs) Within Organoid Systems

CSC-Organoids retain the hierarchical structure of the original tumor, constituting a novel culture system that preserves it. They are also called tumor-initiating cells, which reconstitute the entire cellular heterogeneity of the parental tumor in organoid cultures by proliferating and differentiating into multilineage lineages through self-renewal (Figure 4B) [50]. In organoid cultures, CSCs exist primarily in a quiescent or slow-cycling state. This contributes to the development of therapeutic resistance, as chemotherapeutic drugs are designed to target rapidly proliferating cells [86]. The molecular basis of this resistance has been mainly investigated through organoid studies [87]. For example, gastric cancer organoids have revealed that genes such as KH Domain Containing, RNA Binding, Signal Transduction Associated 3 (KHDRBS3) play a crucial role in acquired resistance to 5-fluorouracil, function as molecular drivers of chemoresistance, and serve as therapeutic targets [88]. With preserved stemness and plasticity, organoids are ready for the identification of CSC markers, the investigation of tolerant cells, and the screening for anti-relapse drugs (Figure 4B) [89].

### 4.3. Tumor Microenvironment Interactions

Organoid platforms successfully replicate the tumor microenvironment (TME), enabling comprehensive modeling of cancer biology. Such models investigate non-epithelial factors, including ECM remodeling, immune effects, nutrient gradients that shape tumor development, and therapeutic efficacy (Figure 4C) [90].

#### 4.3.1. ECM Remodeling

Tumor progression is strongly influenced by biochemical and biomechanical cues from the ECM and CAFs, which modulate niche stiffness and structural architecture in a tumor-type-specific manner [91]. Importantly, to mimic a more native tumor environment, synthetic materials, such as bioengineered 3D hydrogels and porcine lung-derived ECM matrices, have been engineered to recapitulate the biochemical composition and mechanical properties of the in vivo niche [92]. In addition, decellularized extracellular matrix (dECM) derived from native tissues has emerged as an important platform for preserving tissue-specific biochemical cues and structural architecture, thereby enabling more physiologically relevant tumor–matrix interactions [93]. Furthermore, advanced biomaterial scaffolds incorporating functional fragments of ECM proteins, such as integrin-binding ligands and adhesive peptide motifs, have been developed to better replicate cell–ECM signaling mechanisms [94]. Recent studies also employ engineered scaffolds with tunable stiffness, porosity, and surface topology to investigate how mechanical forces and matrix geometry regulate cancer cell invasion, stemness, and therapeutic resistance [94]. These platforms facilitate better representation of tumor-matrix interactions and contribute to our understanding of how ECM dynamics govern invasion, stemness, and drug resistance.

#### 4.3.2. Immune Modulation

Organoids reconstruct the TME through: (1) exogenous immune cell co-culture and (2) air-liquid interface systems that preserve endogenous immunity (Figure 4C) [84]. They offer key advantages for investigating immune checkpoint mechanisms, including Programmed Cell Death Protein 1 (PD-1) and Programmed Death-Ligand 1 (PD-L1), and for testing patient-specific immunotherapy strategies to improve real-world applicability [95,96]. By enabling direct interrogation of tumor–immune crosstalk, immune-competent organoids enhance our understanding of immune evasion and response variability.

### 4.4. Hypoxia and Metabolic Gradients

The 3D organoid structure produces inherent oxygen/nutrient gradients, generating hypoxic and acidic niches typical of solid tumors (Figure 4D) [97]. These gradients make organoids more realistic, but without blood vessels, bigger ones starve for nutrients in the center. To address central starvation, researchers have developed 3D bioprinting and organ-on-a-chip technologies. They enable the formation of perfusable microvascular networks, thereby enhancing the modeling of systemic drug delivery, nutrient exchange, and tumor-circulatory interactions [98]. Such innovations enhance organoid translation by accurately recreating tumor blood flow to tumors, inducing metabolic stress, and improving drug penetration.

## 5. Precision Drug Screening and Therapeutic Selection

**Figure 5:**
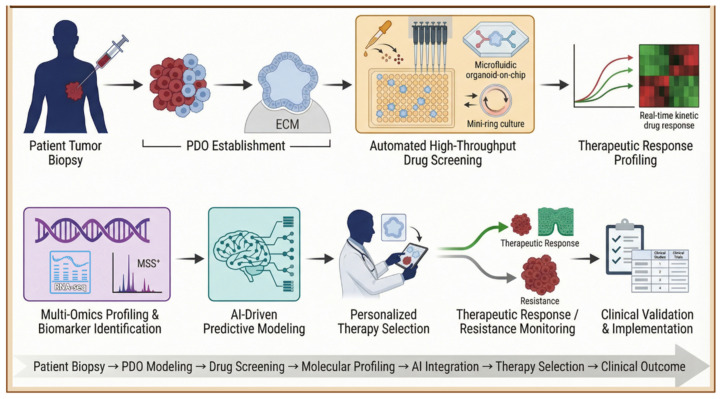
Clinical Workflow for Patient-Derived Organoid (PDO) Precision Medicine and AI-Integrated Therapeutic Selection. This figure illustrates the translational pipeline from patient biopsy to clinical implementation, highlighting the integration of high-throughput technology and computational modeling to guide personalized cancer therapy. Created in BioRender.com (Version 2026) https://BioRender.com/9dmb652 (accessed on 1 March 2026).

Organoids have achieved the highest level of clinical translation through precision drug-screening systems [99]. PDOs mirror accurate ex vivo tumor models for testing drug efficacy and toxicity on an individual basis. This approach avoids the limitations of genome-based stratification, which relies on static molecular changes that do not always predict in vivo drug responsiveness. This functional Precision Medicine approach facilitates individualized therapeutic selection, which is very effective in patients who lack actionable genomic alterations or who have developed resistance to standard treatments (Figure 5) [100].

### 5.1. Organoid-Based High-Throughput Drug Screening

To become clinically and pharmaceutically relevant, organoid platforms must be scalable, standardized, and compatible with high-throughput screening (HTS) [101]. In order to achieve this goal, organoid platforms have been combined with automated robotics and microfluidic systems. However, to improve scalability and reproducibility, automated liquid-handling systems help standardize serial drug dilutions, improve seeding densities, and enhance viability assays, thereby reducing operator-dependent variation [102]. Moreover, culture strategies, such as the “mini-ring” approach, in which cells are seeded around the periphery of a well, leaving an inner area for exchange, enable automated media exchange without manual organoid dissociation or transfer. This setup dramatically reduces turnaround time, enabling actionable drug-response data to be available within 1 week of surgical specimen collection [103]. Furthermore, organoid-on-chip systems in microfluidic devices enable accurate control of both spatial and temporal aspects of drug treatments, including combined treatments and adaptive-dosing schedules [104]. Real-time kinetic analysis identifies treatment schedules that outperform conventional constant-dose monotherapies. These HTS platforms preserve the natural cellular heterogeneity of organoids while letting systematic comparison across multiple patient-derived lines, thereby improving not only biological fidelity but also clinical utility [50].

### 5.2. Predicting Patient-Specific Drug Response

PDOs have high accuracy, sensitivity, and specificity for predicting therapeutic responses [105]. Translational work in metastatic colorectal and gastrointestinal tumors has shown that cell line-based testing with PDOs may achieve positive predictive values ranging from 88 to 100% and negative predictive values of up to 100%, reflecting treatment responses in patients [106]. In advanced colorectal cancer, organoid-based chemotherapy response signatures (also called “chemograms”) were reported to have similar levels of sensitivity and specificity (~75%) and to represent a direct functional decision-support tool in addition to molecular characterization [59]. In contrast to classical preclinical models (2D cell cultures, animal xenotransplantation), PDOs exhibit greater structural conservation and reduced interspecies heterogeneity in predictive drug responses compared with individual patients [107].

### 5.3. Biomarker Identification and Molecular Profiling

Organoid systems have emerged as a valuable assay for biomarker screening and validation [108]. Integrating Pan-omics technologies, such as transcriptomics, quantitative proteomics, genomics, epigenomics, metabolomics, and translatomics, and single-cell sequencing enables comprehensive, high-dimensional mapping of tumor molecular landscapes [109]. Particularly, genomic analyses, such as whole-genome and whole-exome sequencing, enable the identification of driver mutations and structural variants, while epigenomic profiling, including DNA methylation and chromatin accessibility analyses, reveals regulatory mechanisms controlling tumor cell identity and plasticity [110,111,112]. Metabolomic approaches further characterize metabolic reprogramming within tumor organoids, providing insights into altered nutrient utilization and metabolic vulnerabilities associated with cancer progression [113]. In addition, translatomic profiling, such as ribosome-profiling-based approaches, enables the investigation of actively translated mRNAs and post-transcriptional regulation that influence tumor behavior and therapeutic response [114]. Notably, Proteomics-based methods, such as Sequential Window Acquisition of All Theoretical Fragment Ion Mass Spectra (SWATH-MS), have detected reliable protein biomarkers of therapeutic response that better capture functional cellular states compared to RNA signatures, primarily due to post-transcriptional regulatory mechanisms [115]. Additionally, Comparative transcriptomic profiling of healthy versus malignant liver organoids has further identified prognostic indicators linked to unfavorable outcomes in hepatocellular carcinoma [116]. Recent advances in single-cell transcriptomics and integrated single-cell multi-omics approaches further enable the resolution of cellular heterogeneity and lineage dynamics within organoid systems [117]. Such comprehensive datasets now contribute to global efforts such as the Human Cell Atlas (HCA) Organoid Project, which aims to create standardized references for human organoid diversity with direct translational relevance.

### 5.4. Overcoming Therapeutic Resistance

One of the main causes of cancer-related mortality is therapeutic resistance. Organoid models facilitate the investigation of drug resistance mechanisms, including the emergence of drug-tolerant persister (DTP) cell populations, subsets of tumor cells that survive initial therapy without stable genetic mutations [118]. By using serial organoid cultures from the same patient at initial diagnosis, during treatment, and relapse, researchers can employ a sensitive-to-resistant comparative framework to identify changing vulnerabilities and adaptive dependencies [119]. Evidence suggests that CSCs contribute in large part to chemoresistance, and targeting resistance-related genes (e.g., KHDRBS3 in gastric cancer) or pathways, such as Hippo/YAP1, has been shown to overcome resistant phenotypes in organoid models [120]. Furthermore, co-culture systems using CAFs and other stromal components illustrate mechanisms of microenvironment-mediated chemoresistance, where the stromal niches provide protective signals that suppress drug-induced apoptosis, underscoring the importance of modeling tumor–stroma interactions to design more durable therapeutic strategies [121].

### 5.5. Integration with Artificial Intelligence and Multi-Omics Approaches

The introduction of AI and multi-omics technologies has further advanced organoid-based precision oncology by supporting computational analysis of high-content imaging data for organoid morphometry, growth kinetics, and drug response [122]. Additionally, Other AI strategies, including convolutional neural networks implemented in systems such as OrganoIDNet, have facilitated dynamic tracking of organoid development over extensive periods [123]. Moreover, Genomic, transcriptomic, and proteomic data from omics platforms, along with machine learning algorithms, can be used to classify tumor subtypes and predict patient survival more accurately than single-omics modalities [124]. Furthermore, AI-driven organoid systems automate sophisticated data analysis, improve reproducibility, and support real-time predictive modeling, thereby expediting therapy selection, optimizing translational workflows, shortening development cycles, as well as enhancing personalized treatment approaches [125].

### 5.6. Clinical Studies and Trials Utilizing Organoid-Guided Therapy Selection

The translation of PDOs into clinical applications has evolved from retrospective analyses to prospective interventional trials demonstrating proof of concept for functional drug testing in PDOs to guide therapy selection [105]. PDO assays have been used throughout colorectal, gastrointestinal, metastatic, and refractory solid tumors for chemotherapy, targeted therapy, and immunotherapy testing [126]. Important parameters, such as sensitivity, specificity, and the positive predictive value of drug response, indicate strong concordance between organoid responses and patient treatment outcomes, preventing a trial-and-error approach to treating cancer patients [127]. The following (Table 4) summarizes representative clinical studies and trials that have incorporated organoid-guided therapy selection, highlighting cancer type, study design, and key predictive outcomes.

These trials highlight the increasing importance of PDO-guided treatment decision-making from a clinical perspective. The combination of functional drug screening, molecular profiling, AI-assisted analysis, and multi-omics characterization offers attractive opportunities to increase therapeutic precision, reduce treatment inefficiency, and accelerate personalized oncology [134]. While early applications are limited largely to epithelial-derived cancers, recent developments, including trials involving brain tumor organoids, demonstrate the growing extension of organoid platforms to central nervous system malignancies, hematological cancers, and rare tumor subtypes.

## 6. Regenerative Oncology: Dual Role of Stem Cells

**Figure 6:**
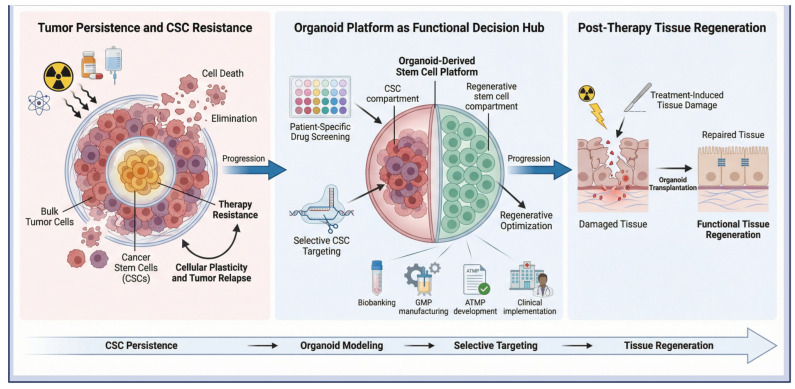
Regenerative oncology model highlighting the dual role of organoid-derived stem cells. The schematic illustrates selective targeting of therapy-resistant cancer stem cells (CSCs) using patient-derived organoid platforms while preserving normal regenerative stem cells. Organoids enable functional drug screening, molecular profiling, and CSC-directed therapies to prevent recurrence. Simultaneously, healthy or engineered organoids support post-therapy tissue repair and functional restoration, bridging tumor eradication with regenerative recovery in precision oncology. Created in BioRender.com (Version 2026); https://BioRender.com/9dmb652 (accessed on 1 March 2026).

Regenerative oncology is an emerging area that emphasizes the two-faced identity and behavior of stem cells in cancer treatment [135]. CSCs turn into cancerous tumor cells, but healthy stem cells participate in the repair of tissues injured by aggressive treatments for malignant disease. So, targeted therapeutic elimination of CSCs is required to prevent tumor recurrence without harming normal stem cells, enabling successful post-treatment tissue repair. In 3D organoid models, we can test drugs that remove tumor cells along with their precursor stem cells, while preserving normal stem cells, which are crucial for regeneration. This strategy will enable researchers to develop therapeutics that not only eradicate the “roots” of cancer but also restore a healthy tissue state [136]. By integrating regenerative engineering with cancer biology, organoid platforms can support pangenomic patient care while reducing side effects (Figure 6) [137].

### 6.1. Targeting Cancer Stem Cells

Cancer treatment is seriously hampered by the existence of CSC, a subset of tumor cells that remain quiescent and resistant to chemotherapy or radiotherapy [138]. Because CSCs constitute a small portion of all cancer cells, common therapies that selectively kill dividing bulk tumor cells and often healthy cells, but cannot effectively eradicate CSCs [139]. That’s why PDTOs have been a key tool in overcoming this resistance, because these models maintain the stemness, hierarchy, and plasticity of tumor cells and closely mimic in vivo. PDTOs are potential models for the discovery of tissue-level CSC markers and drug screening against bulk tumor cells and even resistant CSCs [140]. Recent organoid-based studies have also identified functional plasticity, in which non-CSCs can be redifferentiated to a stem-like state in the tumor microenvironment [141].

### 6.2. Leveraging Regenerative Stem Cell Properties

In addition to disease modeling, organoids are also used to study tissue regeneration by harnessing the innate regenerative capacity of stem cells. Organoids derived from healthy adult tissue or induced iPSCs exhibit physiological and self-renewal properties that mimic tissue architecture, providing a relevant system for studying repair processes [127]. Moreover, iPSC-derived organoids can differentiate into almost all functional tissues and have the potential to serve as an infinite source for organ repair and regeneration [58,128]. Furthermore, these organoids can compensate for interspecies variation in traditional cell culture and animal models, advancing our understanding of human tissue assembly and differentiation [142,143].

### 6.3. Organoids in Post-Therapy Tissue Regeneration

Cancer treatments often cause collateral damage to surrounding healthy tissues. For example, radiation-induced injury to the intestinal epithelium can result in conditions such as radiation proctitis [144]. Organoid technology provides a promising approach for tissue repair and functional reconstruction. For instance, intestinal organoids combined with decellularized matrices have been used to rebuild epithelial structures and “patch” regions damaged by radiation [145]. In preclinical studies, transplantation of healthy colon organoids onto irradiated mucosa restored both epithelial structure and function; these findings indicate that genetically modified organoids could potentially replace or repair organs affected by treatment-induced injury [146].

### 6.4. Organoid Platforms for Cell-Based Therapeutics

The future of personalized oncology is moving toward integrating organoids into advanced therapeutic medicinal products (ATMPs) [147]. Unlike traditional cell therapies, organoids provide a complex multicellular framework capable of functional integration in vivo [136]. “Living biobanks” of organoids can be genetically modified using CRISPR/Cas9 to correct defects or enhance therapeutic properties before autotransplantation [148]. Additionally, 3D bioprinting combined with nanotechnology enables the development of organoid-based biomaterials that can replace or restore tissue function at high resolution [149]. With these advances, organoid-based cell therapeutics are poised to transform clinical practice, offering patient-specific solutions for tissue regeneration and the recovery of biological functions lost due to cancer or its treatment [150].

## 7. Translational and Clinical Applications

Over the years, organoid technology has advanced from a sophisticated in vitro system to a clinically translatable approach. Recent evidence suggests its potential as a diagnostic, drug-discovery, and personalized-therapy tool. The broader use of organoids in basic and translational studies will rely on standards for large-scale production and regulatory compliance to guarantee patient safety, reproducibility, and data reliability [151].

### 7.1. Clinical Trials and Current Implementation

The clinical adoption of organoid technology has expanded rapidly. More than 150 organoid-related studies were registered on ClinicalTrials.gov in 2023, including studies on lung, breast, pancreatic, and colorectal cancers [152]. These studies have validated organoid drug sensitivity against historical patient outcomes and guided real-time organoid screening to individualize therapy [153]. Key applications include selecting neoadjuvant therapies for locally advanced tumors and optimizing salvage treatments for patients at high risk of recurrence [154]. A notable example is a Phase I/II trial in gastrointestinal cancer, in which PDOs achieved 100% sensitivity and 93% specificity in predicting clinical response. Large initiatives, such as TUMOROID and SENSOR, are currently investigating whether organoid-guided therapy can prevent inappropriate administration of novel targeted drugs and improve clinical outcomes [155]. Organoid technology can accurately predict neoadjuvant therapy response in locally advanced tumors and optimize rescue treatment strategies in high-risk patients [156]. Large-scale initiatives such as TUMOROID and SENSOR are currently evaluating whether organoid-guided therapeutic strategies can reduce the unnecessary use of novel targeted agents and improve patient outcomes [157].

### 7.2. Bio Banking and Standardization

Living organoid banks provide access worldwide to traceable, diverse oncogenic tissues for studying the next generation of precision medicine [158]. To ensure the reliability and reproducibility of organoids, biobanking provides ISO-standardized protocols, including safety procedures, documentation, and quality control. However, prolonged culture and repeated subpassaging can lead to loss of intratumoral heterogeneity, clonal selection, and additional mutations, which may cause critical challenges that compromise the biological fidelity of organoid models [50,159]. Moreover, there are some ethical and governance issues, including patient consent, intellectual property, and equitable distribution of commercial benefits [160]. These are critical to maintaining the legitimacy and sustainability of biobanking initiatives.

### 7.3. GMP Production and Scalability

To use Organoid production as advanced therapy medicinal products (ATMPs), it is obvious to follow the Good Manufacturing Practice (GMP) guidelines [161]. These guidelines ensure controlled, sterile production areas; validated, standardized reagents; qualified personnel; and adequate documentation systems that ensure the quality, consistency, and patient safety of the manufactured product. Advances in automated GMP technology support the biobanking-ready production of undifferentiated organoids, such as pancreatic organoids, for large-scale biobanking and clinical translation. Furthermore, Scalability may be further enhanced through automated 3D culture protocols, miniaturized 3D-printable bioreactors (e.g., SpinΩ system), and microfluidic platforms [162]. These platforms reduce batch-to-batch variability, cost reduction, and meet the demands of clinical and industrial applications.

### 7.4. Regulatory Considerations (FDA/EMA Perspectives)

The regulatory landscape is changing due to the unique properties of organoid-based therapies. An important first step in this direction would be the modernization of drug development programs (both preclinical and clinical) by the U.S. FDA, as outlined in its FDA Modernization Act 2.0 [163]. This act is proposed to replace animal studies with the more advanced in vitro models, such as organoids [164]. Because organoids are biological products, they require a Biologics License Application (BLA) or an Investigational New Drug (IND) to demonstrate the safety profile of drugs and clinical trial programs, including process descriptions and pre-clinical safety assessments [165]. Quality control is important not only to the FDA but also to the European Medicines Agency (EMA) for reproducibility, scientific validity, and patient safety. In addition, regulatory authorities are actively developing regulations that address hurdles such as tumorigenic risk and the validation requirement for functional efficacy through Good Laboratory Practice (GLP)-compliant non-clinical studies [166].

### 7.5. Translational Challenges and Proposed Solutions

The modern world is transitioning to therapeutics based on engineered extracellular vesicles, gene-editing platforms, and targeted biologics designed to address molecular disease mechanisms, known as next-generation precision therapeutics. Organoid technology is rapidly developing to transform current treatment platforms and diagnostic methods. In the future, it will transform modern therapeutics as a promising approach to next-generation precision therapeutics [50]. However, numerous biological, technical, regulatory, and logistical obstacles must be overcome to develop and deploy organoid technologies for clinical settings [167]. Solving these challenges is crucial for reproducible, scalable, and clinically actionable organoid models. A summary of the key barriers and strategic response to overcome them is given in Table 5. These interventions help to overcome the biological, technical, regulatory, and ethical barriers to translating organoids. Adoption of these solutions will enable organoids to serve as viable living clinical specimens for drug screening, gene editing, and precision medicine [168].

## 8. Challenges and Future Directions in Organoid-Based Cancer Therapy

### 8.1. Current Limitations and Challenges

Despite their translational potential, organoid-mediated cancer therapies suffer from many biological and technical limitations that prevent their widespread clinical use [176]. A major biological constraint is the lack of intrinsic vasculature and immune components in conventional 3D organoid models. The absence of a functional vascular network impairs nutrient and oxygen diffusion, often leading to necrotic cores in larger organoids [177]. Similarly, the lack of endogenous immune cells and stromal interac-tions doesn’t recapitulate the TME, which is crucial for evaluating immunotherapeutic efficacy and systemic drivers of malignancy [178]. In addition, organoids often fail to reproduce systemic physiological interactions, such as endocrine signaling, metabolic regulation, and multi-organ communication that occur in vivo, limiting their ability to model whole-body tumor progression and metastasis [179].

Additionally, technical challenges further hinder the reproducibility and scalability of organoid systems. However, Culture condition variability remains a significant issue, as animal-derived ECM, such as Matrigel, introduces undefined biochemical stimuli and batch-to-batch variation that can interfere with experimental outcomes [180]. Moreover, differences in media formulations and growth factor compositions across laboratories hamper the implementation of standardized quality control protocols. Another emerging concern is long-term genetic and epigenetic drift during extended organoid passaging, which may alter tumor heterogeneity and reduce fidelity to the original patient tumor [179]. Furthermore, the reagents used for organoid culture are very expensive, and the labor cost are very high, causing a significant obstacle to large-scale production, high-throughput screening, and pharmaceutical applications [101]. Limited automation and the absence of standardized biomanufacturing pipelines further restrict the integration of organoid systems into industrial drug discovery workflows [181]. Ethical and regulatory considerations also add an additional layer of complexity. For example, patient-derived tissues and iPSCs raise concerns about donor privacy, informed consent, and ownership of living biobank resources [173]. In specific disciplines, such as neuro-oncology, the growing complexity of brain organoids has led to ethical discussion on the presence of consciousness and moral status [182]. Furthermore, regulatory uncertainty regarding the classification of organoid-based products, whether as advanced therapy medicinal products (ATMPs), biologics, or research tools, poses additional challenges for clinical translation and commercialization [183].

Patient-to-patient variability also represents an important limitation in PDO research. Differences in tumor stage, tissue composition, and treatment history can affect tissue procurement efficiency, organoid establishment, and long-term culture stability. Additionally, variability in stromal content, immune infiltration, and genetic heterogeneity may lead to inconsistent growth patterns and drug response profiles, making it challenging to establish standardized protocols across laboratories. Another practical challenge is the limited access to patient samples required for PDO generation. Establishing PDOs often depends on collaboration with clinical centers capable of providing fresh surgical or biopsy tissues and maintaining biobanking infrastructure. Consequently, many laboratories may not have equal access to these platforms. Expanding tissue-sharing networks, standardized biobanks, and international collaborations will be essential to ensuring broader, more equitable access to PDO technologies.

However, most current clinical and preclinical applications still concentrate on gastrointestinal and hematological malignancies, and organoid platforms from other solid tumors remain comparatively underexplored [15]. Future progress will require improved vascularized and immune-competent organoid systems, integration with microfluidic organ-on-chip technologies, standardized culture platforms, and AI-driven analysis pipelines to enhance reproducibility and translational relevance [184,185,186]. By establishing comprehensive frameworks that define and classify organoids as biological products with safety and quality standards, we can overcome these constraints.

### 8.2. Emerging Technological Solutions and Future Directions

Overcoming the challenges posed by organoid technology requires integrating bioengineering innovations, high-resolution molecular mapping, and AI. Integrating these technologies may develop organoid platforms that are both physiologically relevant and reproducible [187]. A cutting-edge approach is the Microfluidic organoid-on-chip systems that incorporate vascular networks, immune cells, and dynamic flow conditions to precisely replicate in vivo physiology [188]. Additionally, these systems also replicate physiologic shear stress and permit recruitment of circulating immune cells, providing a functional in vitro niche better suited for drug testing and disease modeling. Furthermore, the 3D-bioprinting system enables the architectural construction of organoids using modifiable materials to generate complex tissue-like 3D architectures with vascularization and spatial organization [189]. Genetic engineering and multi-omics further enhance the potential organoid. For instance, CRISPR-based genome editing can precisely introduce oncogenic mutations and repair genetic lesions in human-derived organoids [190]. Moreover, in combination with spatial transcriptomics and multi-omics approaches, these platforms result in a high-resolution molecular map of the cellular composition, interactions, and functional states of an organoid niche. These comprehensive profiling efforts ensure detailed characterization of intratumoral heterogeneity and an understanding of the mechanisms of drug resistance. AI is mandatory to optimize the analysis of the large datasets generated by these advanced organoid platforms. Machine learning approaches will also pave the way for automated drug screening [191]. They enable personalized prediction of therapeutic response, and decision of treatment selection, unbiased and reproducible to a far greater extent than any manual interpretation [192]. These AI technologies and models are able to monitor macro- and micro-scale architectural features and real-time organoid response, which come together to offer high-content readout datasets for personalized therapeutic decisions in the future.

## 9. Conclusions

Organoids have revolutionized oncology by enabling personalized care for individual cancer patients by accurately mimicking tumor physiology, genetic heterogeneity, and morphological architecture, enabling precise investigation of cell-autonomous and non-cell-autonomous pathways to cancer progression and therapy resistance. Conventional 2D cultures or preclinical models cannot fully mimic tumor physiology due to interspecies differences in genetics, immune systems, biochemistry, and TME composition. Various studies & clinical trials have demonstrated that patient-derived organoids (PDOs) achieve more than 90% sensitivity and specificity in predicting therapeutic responses, thereby reducing the chance of therapeutic failures. The organoid platform also brought regulatory changes & acts, such as the FDA Modernization Act 2.0, which aims to enable the replacement to animal testing soon, saving animal lives and minimizing biosafety risks. However, organoid platforms have several major limitations, including vascularization, nerve innervation, immune cell incorporation, and tumor microenvironmental complexity. Moreover, the standardization of culture protocols, reagent cost, batch-to-batch variability, limited long-term stability, patient privacy & biobank ownership are also some unsolved concerns. In the future, integrating bioengineering, multi-omics profiling, and AI-driven analytics is expected to address most of these limitations, while organoid-on-a-chip systems, 3D bioprinting, and GMP-scale production will enable more reproducible, high-throughput applications. Taken together, Organoid technology will be the future of precision oncology and regenerative medicine, ensuring personalized cancer therapies and the reduction in worldwide mortalities due to cancer.

## Figures and Tables

**Table 1:** Core Signaling Pathways Regulating Organoid Development and Cancer Pathogenesis.

Signaling Pathway	Role in Organoid Development	Role in Cancer Biology & Pathogenesis	Key Niche Modulators (In Vitro)	References
Wnt/β-catenin	Master regulator of stem cell maintenance, proliferation, polarity, and crypt–villus axis formation. Essential for sustaining Lgr5^+^ intestinal stem cells and long-term organoid expansion. Maintains epithelial identity and structural integrity.	Aberrant activation drives carcinogenesis in colorectal, breast, esophageal, and hepatocellular cancers. Promotes tumor initiation, EMT, metastasis, and maintenance of cancer stem cells (CSCs). Frequently mutated (e.g., APC, β-catenin).	R-spondin-1 (Lgr5 ligand), Wnt3a, CHIR99021 (GSK3β inhibitor), LGK974 (Porcupine inhibitor)	[39,40]
Notch	Regulates stem cell preservation versus lineage differentiation through lateral inhibition. Maintains proliferative epithelial progenitors and suppresses secretory lineage differentiation in intestinal and epithelial organoids.	Supports tumor maintenance, EMT, angiogenesis, and chemoresistance. Dysregulated in breast, gastric, pancreatic, and hematologic malignancies. Promotes CSC survival and therapy resistance.	Jagged-1, DLL4 (ligands), DAPT (γ-secretase inhibitor), RO4929097	[41,42]
Hedgehog (Hh)	Controls embryonic patterning, morphogenesis, epithelial–mesenchymal interactions, and tissue regeneration following injury. Modulates stem cell proliferation and differentiation gradients.	Aberrant activation contributes to tumor initiation and progression in lung, basal cell carcinoma, pancreatic, and GI cancers. Regulates CSC self-renewal and resistance (e.g., Sorafenib resistance).	Sonic Hedgehog (SHH), Purmorphamine (agonist), Cyclopamine, GANT61 (GLI inhibitor)	[43]
TGF-β/BMP	BMP restricts stem cell overexpansion and promotes differentiation. TGF-β regulates organoid initiation, epithelial integrity, and tissue homeostasis. Balances proliferation and lineage commitment.	Exhibits context-dependent duality. Tumor-suppressive in early carcinogenesis; pro-metastatic in advanced disease via EMT induction, invasion, immune evasion, and stromal remodeling.	Noggin (BMP inhibitor), A83-01 (ALK4/5/7 inhibitor), SB431542, Recombinant TGF-β1	[44]
Hippo/YAP/TAZ	Regulates organ size, regeneration, and epithelial repair. YAP/TAZ act as mechanosensitive transcriptional co-activators integrating ECM stiffness and architectural cues. Nuclear exclusion suppresses proliferation in differentiated cells.	Persistent YAP/TAZ activation drives solid tumor initiation, metastasis, metabolic reprogramming, and CSC expansion. Interacts with Wnt and TGF-β signaling to enhance tumorigenicity.	XMU-MP-1 (MST1/2 inhibitor), Verteporfin (YAP inhibitor), Y-27632 (ROCK inhibitor)	[45]
PI3K/AKT/mTOR	Supports metabolic homeostasis, protein synthesis, survival, and proliferation in 3D organoid cultures. Coordinates growth factor responses and interacts with Wnt and Hedgehog pathways.	Frequently hyperactivated in breast, lung, colorectal, and glioblastoma cancers. Promotes CSC survival, metabolic adaptation, therapeutic resistance, and hyperproliferation.	Insulin, IGF-1, EGF, Rapamycin (mTOR inhibitor), LY294002 (PI3K inhibitor)	[44,46]
JAK/STAT	Regulates stem cell survival, self-renewal, and differentiation, particularly in inflammatory and stromal contexts. Influences cancer-associated fibroblast (CAF) heterogeneity in organoid co-cultures.	Promotes tumor growth, immune evasion, invasion, and metastasis, particularly in lung and colorectal cancers. Activated by inflammatory cytokines (e.g., IL-6), linking inflammation to tumor progression.	IL-6, LIF, HGF, Ruxolitinib (JAK inhibitor)	[47]
MAPK/ERK	Mediates responses to growth factors, regulating proliferation, migration, and differentiation. Essential for organoid growth and niche responsiveness to EGF/FGF gradients.	Constitutive activation (e.g., KRAS, BRAF mutations) drives uncontrolled proliferation, metastasis, and drug resistance in multiple cancers.	EGF, FGF2, FGF7, FGF10, MEK inhibitors (U0126, Trametinib)	[48]
NF-κB	Regulates cell survival, inflammatory signaling, and differentiation during tissue development. Coordinates immune–epithelial interactions in organoid co-cultures.	Promotes tumor-associated inflammation, CSC survival, chemoresistance, and microenvironment remodeling in GI and breast cancers.	TNF-α, LPS, BAY 11-7082 (NF-κB inhibitor)	[49]

**Table 2:** Comparative overview of preclinical cancer modeling platforms.

Feature	2D Cell Culture	Animal Models (PDX/GEMM)	Patient-Derived Tumor Organoids (PDTOs)	iPSC-Derived Organoids	ASC-Derived Organoids	References
Architecture & Structural Complexity	Simple monolayer; lacks 3D architecture, polarity, and tissue organization.	Highly complex in vivo systems include systemic interactions and vascularization.	3D self-organizing structures preserving tumor histoarchitecture.	Complex 3D multicellular structures reflecting developmental programs.	3D epithelial structures resembling mature tissue units.	[50]
Establishment Success Rate & Timeframe	High success rate; rapid growth (days).	Variable engraftment; long latency (months).	Moderate–high success; expansion within weeks.	High long-term expandability; differentiation requires weeks–months.	High establishment efficiency; moderate expansion time.	[51]
Preservation of Genetic & Phenotypic Heterogeneity	Limited; selective for rapidly proliferating clones.	High preservation of intratumoral heterogeneity.	High retention of genomic, transcriptomic, and phenotypic traits.	Initially low tumor heterogeneity; genetically engineerable (e.g., CRISPR).	High preservation of tissue-specific molecular signatures.	[52]
Tumor Microenvironment (TME) Representation	Absent; no stromal or immune components.	Fully present; includes vasculature and host-derived immune/stromal cells.	Partial; can be enhanced via immune/stromal co-culture.	Can be engineered to incorporate multiple lineages; limited intrinsic TME.	Primarily epithelial; stromal co-culture possible.	[53]
Cost & Ethical Considerations	Low cost; minimal ethical concerns.	Very high cost; significant regulatory and ethical oversight.	Moderate cost; ethical considerations for patient tissue procurement.	High derivation cost; fewer ethical concerns than embryonic stem cells.	Moderate cost; dependent on tissue accessibility.	[54]
Scalability & High-Throughput Screening	Highly compatible with automated high-throughput screening.	Limited scalability; unsuitable for large-scale screening.	Compatible with medium- to high-throughput drug screening.	Scalable expansion; suitable for developmental and pharmacological screening.	Suitable for drug and toxicity testing; moderate scalability.	[55]
Species-Specific Relevance	Human-derived but phenotypically altered in 2D conditions.	Subject to interspecies physiological differences.	Fully human-derived; high fidelity to patient biology.	Human-derived; avoids species-related bias.	Human-derived; mimics native epithelial responses.	[56]
Genetic Manipulation Feasibility	Highly amenable to gene editing and transfection.	Genetic modification possible (GEMMs) but complex and time-consuming.	CRISPR editing feasible but technically demanding.	Highly amenable to genome editing prior to differentiation.	Gene editing possible; efficiency varies by tissue type.	[57]
Clinical Predictive Value	Limited predictive accuracy for clinical response.	Moderate translational predictability; species limitations remain.	High predictive potential for therapy response and resistance profiling.	Valuable for modeling early tumorigenesis and hereditary cancer.	Useful for modeling early mutational events and tissue-specific tumor initiation.	[58]

**Table 3:** Multi-component organoid platforms for modeling tumor–microenvironment interactions.

Model Type	Integrated Components	Biological Purpose	Key Applications	Advantages	Limitations	References
Immune-Organoids	Tumor organoids co-cultured with T cells, NK cells, macrophages, dendritic cells (autologous or allogeneic)	Recapitulation of tumor–immune crosstalk within an immunocompetent microenvironment	Immunotherapy testing (immune checkpoint blockade, CAR-T cytotoxicity), immune evasion studies, and resistance mechanisms	Enables patient-specific immune response modeling; supports precision immunotherapy development	Limited long-term immune cell viability; incomplete systemic immune complexity	[70]
Vascularized Organoids	Tumor organoids with endothelial cells or mesodermal progenitors; perfusable microvascular networks via bioprinting or organ-on-chip	Modeling angiogenesis, nutrient/oxygen diffusion, tumor–circulatory interactions	Drug penetration studies, metastasis modeling, and angiogenesis research	Improves physiological relevance; allows perfusion-based assays and systemic drug distribution modeling	Technical complexity; variability in stable vascular network formation	[71]
Tumor–Stroma Models	Tumor organoids co-cultured with cancer-associated fibroblasts (CAFs), endothelial cells, and ECM components	Reconstitution of stromal support and tumor-permissive niches	Investigation of invasion, metastasis, drug resistance, and stromal targeting strategies	Captures bidirectional tumor–stroma communication; identifies stromal therapeutic targets	Stromal composition may not fully reflect in vivo heterogeneity	[72]

**Table 4:** Representative clinical studies and trials utilizing organoid-guided therapy selection.

Organ System/ Cancer Type	Trial Name	NCT ID	Study Design/Phase	Sample Size (*n*)	Key Findings/ Predictive Concordance	Status	References
Gastro- intestinal Cancers	Target CRC	NCT05401318	Observational	40	Identifies chemotherapy combinations and targeted therapies to induce immunotherapy efficacy	Recruiting	[128]
	Pancreatic Adjuvant	NCT04931381	Interventional	100	Adjuvant chemotherapy selection guided directly by PDO drug sensitivity testing	Recruiting	[129]
	Gastric Neoadjuvant	NCT06196554	Observational	40	Evaluates the inconsistency between organoid oxaliplatin screening and actual clinical neoadjuvant response	Recruiting	[128]
	Organoids-on-a-chip	NCT04996355	Observational	52	Validates the accuracy and sensitivity of microfluidic “on-chip” drug screening for advanced CRC	Recruiting	[128]
	Biliary Tract Study	NCT04561453	Interventional	20	Combines multi-platform profiling with PDO drug sensitivity and ctDNA monitoring	Recruiting	[129]
	Consistency Study	NCT06100016	Observational	105	Large-scale consistency assessment of novel PDO drug susceptibility testing methods	Recruiting	[128]
Multi-Organ/Pan-Cancer Studies	Multi-Cancer Guide	NCT04931394	Interventional	200	Large-scale trial using PDOs to guide chemotherapy selection across multiple cancer types	Recruiting	[130]
	Precision Feasibility	NCT03952793	Correlative	—	Multicenter study investigating the feasibility and predictive accuracy across diverse hospital settings	Active	[131,132]
Breast Cancer	Metastatic HER2- BC	NCT05024734	Interventional	33	Chemotherapy selection guided by PDO sensitivity testing	Recruiting	[130]
CNS Tumors	Brain Organoid Precision Study	NCT06781372	Observational	—	Evaluates feasibility of patient-derived brain tumor organoids for predicting individualized therapeutic responses	Recruiting	[133]

**Table 5:** Comprehensive Translational Challenges and Proposed Solutions in Organoid Technology.

Challenges	Key Obstacles	Proposed Solutions & Strategic Directions	References
Biological Fidelity	Genetic drift and clonal selection: Progressive loss of intratumoral heterogeneity during extended passaging. Stromal deficiency: Limited representation of native immune cells, fibroblasts, and extracellular matrix components.	Incorporate autologous immune and stromal co-cultures using air–liquid interface systems or microfluidic platforms to better recapitulate the tumor microenvironment. Preferentially use early-passage organoids to maintain genomic integrity and tumor fidelity.	[169,170]
Technical & Scalability Constraints	Nutrient and oxygen gradients: Development of hypoxic cores and necrosis in large organoids. Low efficiency and high costs: Expensive growth factors and labor-intensive manual protocols limit scalability.	Engineer perfusable vascular networks through 3D bioprinting and organ-on-chip systems to improve nutrient delivery. Adopt automated high-throughput liquid handling platforms and mini-ring culture techniques to enhance reproducibility and reduce cost.	[171]
Standardization & Reproducibility	Inter-batch variability: Differences across patient-derived samples and laboratory conditions. Lack of universal quality metrics: Absence of standardized criteria to assess tumor fidelity and functional equivalence.	Develop ISO-aligned standardized operating procedures for histopathological validation, genomic and transcriptomic profiling, and niche-dependency assays. Utilize well-characterized reference organoid lines as benchmarking controls.	[172]
Ethical & Social Considerations	Informed consent complexity: Challenges in obtaining broad, future-use consent from donors. Commercialization concerns: Issues related to donor privacy, intellectual property, and equitable benefit sharing.	Establish transparent governance frameworks addressing donor rights, data ownership, and intellectual property distribution. Implement secure, de-identified, and encrypted data infrastructures to facilitate responsible collaboration.	[173]
Regulatory & Legal Uncertainty	Product classification ambiguity: Unclear regulatory status as biological products, advanced therapeutic medicinal products (ATMPs), or research tools. Translational validation challenges: Demonstrating safety and efficacy without reliance on traditional animal models.	Harmonize development strategies with FDA/EMA regulatory pathways and the Modernization Act 2.0 framework. Perform GLP-compliant non-clinical validation studies to support translational advancement.	[174]
Logistical & Resource Limitations	Cryopreservation difficulties: Reduced viability and structural integrity after freezing mature 3D organoids. High media and matrix costs: Dependence on expensive, chemically defined media and Matrigel-based scaffolds.	Optimize vitrification and controlled slow-freezing protocols tailored to 3D structures. Develop cost-effective synthetic or defined biomaterial scaffolds to replace Matrigel and reduce long-term expenses.	[175]

## Data Availability

No new data were created or analyzed in this study. Data sharing is not applicable to this article.
